# Crystal Guava (*Psidium guajava* L. “Crystal”): Evaluation of *In Vitro* Antioxidant Capacities and Phytochemical Content

**DOI:** 10.1155/2020/9413727

**Published:** 2020-09-01

**Authors:** Rika Hartati, Hashifah I. Nadifan, Irda Fidrianny

**Affiliations:** Department of Pharmaceutical Biology-School of Pharmacy, Bandung Institute of Technology, Bandung 40132, Indonesia

## Abstract

Free radicals can cause many diseases, such as cancer. Antioxidant is a compound that could scavenge free radicals. One of the natural antioxidants is guava. The goals of this research were to investigate the antioxidant activity of leaves and fruit of crystal guava by determining the value of the Antioxidant Activity Index (AAI) using DPPH, CUPRAC, and FRAP; evaluate the total phenolic content (TPC) and total flavonoid content (TFC); analyse the correlation between the TPC and TFC with AAI DPPH, CUPRAC, and FRAP, and analyse the correlation between the 3 methods. Extraction was performed by the reflux method using n-hexane, ethyl acetate, and ethanol. Determination of AAI DPPH, CUPRAC, FRAP, the TPC, and the TFC was performed by UV-visible spectrophotometry. The correlation of the TPC and TFC with AAI DPPH, CUPRAC, and FRAP and, also, the correlation of the 3 methods were investigated by Pearson's method. The antioxidant activity of leaves and fruit extracts of crystal guava showed AAI DPPH in the range of 0.33–56.46, CUPRAC 0.20–7.31, and FRAP 1.65–59.89. The highest TPC was given by ethanol leaf extracts (49.55 ± 1.45 g GAE/100 g), while the highest TFC was for n-hexane leaf extracts (9.68 ± 0.210 g QE/100 g). The TPC of leaves extract had a significantly positive correlation with AAI DPPH, CUPRAC, and FRAP. AAI DPPH, AAI CUPRAC, and AAI FRAP of leaves and fruit extract of crystal guava showed a significantly positive correlation. In general, leaves extract had strong antioxidant activity by the three methods. For the highest antioxidant activity, ethanol was the best solvent for extraction leaves and ethyl acetate for extraction fruit of crystal guava. The TPC in leaves extract contributed to the antioxidant activity by DPPH, CUPRAC, and FRAP methods. The Antioxidant activity of leaves and fruit extracts of crystal guava was linear by the three methods.

## 1. Introduction

Free radical is a small molecule that is very reactive because of its unpaired electron [1]. Antioxidant is a compound that could scavenge a free radical [2]. In our body, there is a balance between the antioxidant and free radical. The imbalance of a free radical with an antioxidant can cause various diseases such as cancer, hypertension, and diabetes. Consumption of an antioxidant can prevent the effect from a free radical. Because when an antioxidant reacts with a free radical, it can produce a more stable compound and does not cause disease in the body. One of the natural antioxidants is guava. Crystal guava is the one of the most favorite guava in Indonesia because of its crispy fruit flesh and seedless fruit. Antioxidant properties of crystal guava in Indonesia have not been studied.

DPPH (2,2-diphenyl-1-picrylhydrazyl), ABTS (2,2′-Azino-bis (3-ethylbenzothiazoline-6-sulfonic acid), CUPRAC (Cupric ion-Reducing Antioxidant Capacity), and FRAP (Ferric Reducing Antioxidant Power) methods could be applied to observe the antioxidant activity in many plant extracts [3–5]. Previous studies [6–12] exposed that DPPH, CUPRAC, and FRAP methods could evaluate antioxidant activities of *Psidium guajava*. The phenolic content in a plant might be linked with its antioxidative properties [6]. *Psidium guajava* contained a phenolic compound [3] and flavonoids such as apigenin, quercetin, and morin in its leaves [13]. Fruit of guava also had high ascorbic acid content, whose ascorbic acid has antioxidant activity.

There are many extraction methods, such as reflux, continuous extraction using the Soxhlet apparatus, and maceration. In this research, extraction was performed by reflux using increasing polarity of solvents, n-hexane, ethyl acetate, and ethanol, consecutively. These three solvents were used to compare the antioxidant activity from a nonpolar compound (n-hexane extract), semipolar compound (ethyl acetate extract), and polar compound (ethanol extract) of leaves and fruit of crystal guava. The purposes of this research were to investigate the antioxidant profile of leaves and fruit of crystal guava (“*Psidium guajava* L.” “crystal”) by determining the Antioxidant Activity Index (AAI) using DPPH, CUPRAC, and FRAP; determining the total phenolic content (TPC) and total flavonoid content (TFC); analysing the correlation of the TPC and TFC with AAI DPPH, CUPRAC, and FRAP, and analysing the correlation between the 3 methods.

## 2. Materials and Methods

### 2.1. Materials

Gallic acid, quercetin, DPPH (2,2-diphenyl-1-picrylhydrazyl), neocuproine, and 2,4,6-tripyridyl-S-triazine (TPTZ), obtained from Sigma-Aldrich, ferric chloride, and cupric chloride. Other reagents were of analytical grades and obtained from Merck.

### 2.2. Preparation of the Sample

Two organs from *Psidium guajava* L. “crystal” that were leaves named as LV and fruit as FR were obtained from Cianjur, West Java-Indonesia. The sample was cleaned with water, for sortation from contaminant, cut, dried, and milled into a powder. The drying process was carried out in a drying cabinet at 45–50°C. The milling process was performed by using a knife mill grinder equipment, and 20 mesh size of powder was obtained.

### 2.3. Extraction of Leaves and Fruit of Crystal Guava

Extraction was performed by reflux using increasing polarity of solvents, n-hexane as the nonpolar solvent, ethyl acetate as the semipolar solvent, and ethanol as the polar solvent, consecutively. Three hundred grams of the powdered sample was applied. Each solvent was repeated three times. Thus, there were n-hexane leaves extract (LV1), n-hexane fruit extract (FR1), ethyl acetate leaves extract (LV2), ethyl acetate fruit extract (FR2), ethanol leaves extract (LV3), and ethanol fruit extract (FR3). In one time extraction, the process was performed in 2-3 hours after the solvent boiled.

### 2.4. Determination of the Total Phenolic Content (TPC)

The Folin–Ciocalteu reagent [14], with slight modification, was utilized in the determination of the total phenolic content. Analysis was conducted in triplicate for each extract. The wavelength of observation was 765 nm. The calibration curve of gallic acid was running at a concentration of 40–130 *μ*g/ml. The TPC was presented as gallic acid equivalent per 100 g extract (g GAE/100 g). Each extract and gallic acid dissolved in methanol. A sample of 0.5 mL was mixed with 5 mL Folin–Ciocalteu 10% and 4 mL sodium carbonate 1 M. The mixture was incubated 15 min, and the absorbance was investigated by using a UV-Vis spectrophotometer.

### 2.5. Determination of the Total Flavonoid Content (TFC)

Quercetin 60–130 *μ*g/ml was applied to get a calibration curve. The absorbance was investigated at 415 nm. Chang et al.'s [15] method was performed to determine the TFC and expressed as quercetin equivalent per 100 g of extract. Analysis was triplicate for each extract. Each extract and quercetin dissolved in methanol. A sample of 0.5 mL was mixed with 1.5 methanol, 0.1 mL aluminum (III) chloride 10%, 0.1 mL sodium acetate 1 M, and 2.8 mL water. The mixture was incubated for 30 min at room temperature, and the absorbance was read.

### 2.6. Determination of the AAI (Antioxidant Activity Index) of DPPH

Blois's method [16] with some modification was applied in the determination of the antioxidant activity by DPPH. Each extract was processed in various concentrations and mixed with the DPPH solution of 39.4 *μ*g/ml (volume 1 : 1). The DPPH solution was prepared in methanol. The absorbance was evaluated at 515 nm by UV-vis spectrophotometry, after 30 min of incubation. The standard was ascorbic acid. Analysis was carried out in triplicate. The purple color of the DPPH solution will be shifted when the free radical was scavenged by antioxidant, so IC_50_ (inhibitory concentration 50%) can be determined from the sample. A calibration curve was plotted between % of DPPH scavenging activity versus concentration, to obtain IC_50_. Thereafter, the AAI of each extract was determined by dividing the final concentration of DPPH with IC_50_ at each extract.

### 2.7. Determination of the AAI of CUPRAC

Several concentrations of extract were added into the CUPRAC solution of 100 *μ*g/mL (volume 1 : 1) [17]. After incubation for 30 min, the absorbance was investigated at a wavelength of 450 nm. The CUPRAC solution was prepared by mixing CuCl_2_ in aquadest and neocuproine in ethanol, and then, the mixture was dissolved in ammonium acetate buffer pH 7. Ascorbic acid was applied as standard. Analysis was performed in trireplication. Cu (II) will reduce to Cu (I) if the sample acts as an antioxidant. Neocuproine's function is to make a chromophore with Cu (I) and show a yellow color, and then, the exhibitory concentration 50% (EC_50_) was obtained from the calibration curve. Afterwards, by dividing the final concentration of CUPRAC with EC_50_, the AAI of each extract can be calculated.

### 2.8. Determination of the AAI of FRAP

The procedure from Benzie and Strain with minor modification was utilized to process the FRAP solution [18]. The FRAP solution was prepared by mixing TPTZ in HCL and FeCl_3_ in aquadest, and then, the mixture was dissolved in acetate buffer pH 3.6. Various concentrations of each extract was set up and pipetted into the FRAP solution 467.5 *μ*g/ml (volume 1 : 1). The standard was ascorbic acid. Evaluation was performed at 593 nm, after 30 min of incubation. Triplicate analysis was conducted. Reduction of Fe (III) to Fe (II) will occur when the sample acts as an antioxidant, and the Fe (II)-TPTZ complex will show a blue color. The value of exhibitory concentration 50% (EC_50_) can be observed by the calibration curve. Then, the AAI of each extract was calculated using the final concentration of FRAP divided by EC_50_ at each extract.

### 2.9. Calculation of the Antioxidant Activity Index (AAI)

The DPPH scavenging activity, CUPRAC capacity, and FRAP capacity of crystal guava extracts were denoted as the AAI. The estimation of the AAI was performed with the following equation:

(1) $$\begin{array}{c} \text{AAI}=\frac{\text{ final concentration of radical solutions}\left(\mu \text{g}/\text{ml}\right)}{\text{ IC}50\text{ or EC}50\;\left(\mu \text{g}/\text{ml}\right)}. \end{array}$$

### 2.10. Statistical Analysis

Statistical analysis was carried out by SPSS 25 for Windows. Analysis of each sample was in trireplication. All of the expressed results were means ± standard deviation. The statistical significance was observed using the one-way ANOVA method (*p* < 0.05).Pearson's correlation method was applied to analyse the correlation of the TPC, TFC, and the antioxidant activity and between antioxidant activity testing methods.

## 3. Results and Discussion

Previous research [6–12] reported that *Psidium guajava* had antioxidant capacity. There was no research concerning the antioxidant capacity of different extracts (n-hexane, ethyl acetate, and ethanol) of leaves and fruit from crystal guava (“*Psidium guajava* L.”“crystal”) which is grown in Cianjur, West Java-Indonesia, using three methods (DPPH, CUPRAC, and FRAP).

### 3.1. AAI of Leaves and Fruit Extracts from Crystal Guava

DPPH is a stable free radical, whose absorbance can be read at 517 nm. The purple color of the original DPPH will be shifted when the free radical is scavenged by an antioxidant. The decrease in the absorbance of DPPH is connected to the antioxidant potential of the sample [19]. Neocuproine is a reagent of CUPRAC, which was combined with cupric chloride in ammonium acetate buffer pH 7, and Cu (II) will reduce to Cu (I), if the sample acts as an antioxidant. Neocuproine's function is to make a chromophore with Cu (I) and show a yellow color, which can be read at 450 nm [20]. A sample which can act as a reductor and be oxidized by Cu (II) if its reduction potential is lower than 0.159 V, reduction potential of Cu (II)/Cu (I) [21]. The reagent of FRAP is a combination of ferric (III) chloride and TPTZ in acetate buffer pH 3.6. Reduction of Fe (III) to Fe (II) will occur when the sample acts as an antioxidant. The Fe (II)-TPTZ complex will show a blue color, and the absorbance at 593 nm can be evaluated. The reduction potential of the antioxidant sample should be lower than the reduction potential of Fe (III)/Fe (II) [20].

Before using a method, it should be verified. The verification process of the method in the present study had been performed applying ascorbic acid as standard. The AAI of ascorbic acid was 29.4561 ± 1.573 by DPPH, 92.911 ± 4.526 by FRAP, and 21.988 ± 1.8726 by CUPRAC methods. After the methods were verified, the sample was tested by three methods. The AAI of DPPH, CUPRAC, and FRAP in leaves and fruit extract from crystal guava is shown in Table 1.

IC_50_ of DPPH scavenging capacity is the concentration of the sample or standard that can reduce 50% absorbance of DPPH. Meanwhile, EC_50_ of CUPRAC or FRAP capacity is the concentration of the sample or standard that can increase 50% absorbance of CUPRAC or FRAP capacity. The highest antioxidant capacity means had the lowest IC_50_ or EC_50._ But, when the concentration of the radical solution was different, the result of IC_50_ or EC_50_ could be different too, while the AAI would be similar [22]. According to the AAI, the antioxidant activity could be classified into four groups, poor (AAI < 0.05), moderate (0.5 ≤ AAI < 1), strong (1 ≤ AAI ≤ 2), and very strong (AAI > 2) [22].

Extraction was performed using n-hexane, ethyl acetate, and ethanol, consecutively. The nonpolar solvent was n-hexane, which will selectively extract only nonpolar compounds, and it led to just nonpolar compounds contained in n-hexane extract. Meanwhile, the semipolar solvent which was ethyl acetate, which will extract a small portion of nonpolar compounds and most of the semipolar compounds. Because most of the nonpolar compounds have been extracted by n-hexane, ethyl acetate extract mostly contained semipolar compounds. Then, finally, extraction was performed with ethanol, in which a polar solvent will extract a small portion of nonpolar and semipolar compounds and also extract mostly polar compounds. Hence, with this method, we will get the highest yield of nonpolar, semipolar, and polar compound extract, and then, the antioxidant activity could be compared. Table 1 displays that extraction by using increasing polarity can affect the antioxidant activity with CUPRAC and FRAP methods. There were significant differences among the AAI of three solvents in leaves and fruit extracts.

In the present research, the AAI of DPPH from leaves and fruit extracts of crystal guava varied in the range of 0.33–56.46. The highest AAI DPPH was shown by ethanol leaves extract (LV3) 56.46, followed by ethyl acetate leaves extract (LV2) 1.825 and n-hexane leaves extract (LV1) 1.00, while the ascorbic acid standard expressed an AAI DPPH 29.45. Meanwhile, when using the CUPRAC method, we figured various results in the range of 0.20–7.31. The highest AAI CUPRAC was given by ethanol leaves extract (LV3) 7.31, followed by ethyl acetate leaves extract (LV2) 2.84 and ethyl acetate fruit extract (FR2) 1.51, while the ascorbic acid standard had an AAI CUPRAC 21.98. In the current study, the AAI of FRAP ranged from 1.65 to 59.89. The highest AAI FRAP was observed in ethanol leaves extract (LV3) 59.89, followed by ethyl acetate fruit extract (FR2) 37.40 and ethyl acetate leaves extract (LV2) 17.41, while the ascorbic acid standard showed an AAI FRAP 92.91.

This research revealed that, in general, ethanol and ethyl acetate leaves extract from crystal guava had an AAI more than 2 using DPPH, CUPRAC, and FRAP methods, which was clustered as a very strong antioxidant. Ramadhania et al. [12] showed that methanol leaves extract had the highest antioxidant activity by the DPPH method, followed ethyl acetate and n-hexane extract, with polarity solvent extraction. The study by Cedric et al. [10] also reported that methanol leaves extract of *P*. *guajava* had the highest antioxidant activity by DPPH and FRAP methods, followed by ethyl acetate and n-hexane extracts. Their research [10, 12] was similar with our study, as ethyl acetate had higher antioxidant activity than n-hexane extract. Manikandan et al. [8] showed that ethanol leaves extract had the highest antioxidant activity using the DPPH method than methanol and water extract, by Soxhlet extraction. The study by Bouchoukh et al. [6] demonstrated that guava leaves from Algeria had the highest antioxidant activity in ethyl acetate extract, when compared to butanol and chloroform extracts, using the DPPH method, while with the CUPRAC method, the highest antioxidant activity was given by butanol [6]. The study by Ashraf et al. [7] using guava from Pakistan showed that n-hexane leaves extract had the lowest antioxidant activity, compared to methanol and chloroform extracts, using DPPH and FRAP methods. Similar to this research, Zahin et al. [9] reported that the antioxidant activity of ethanol leaves extract of *P*. *guajava* was always higher than that of ethyl acetate extract using DPPH, CUPRAC, and FRAP methods. The study by Liu et al. [11] using white guava, with DPPH, FRAP, and ABTS methods, presented that the highest antioxidant activity was found in peel extract, followed by flesh and seed extract, respectively.

### 3.2. TPC and TFC in Leaves and Fruit Extracts from Crystal Guava

**Figure 1:**
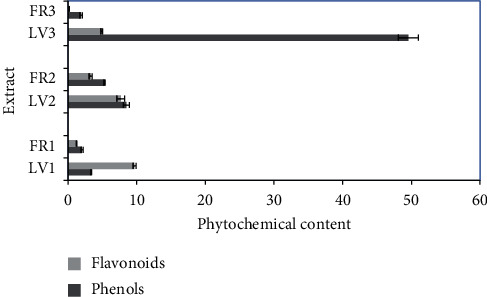
Phytochemical content in crystal guava extracts, LV = leaves, FR = fruit, 1 = n-hexane extract, 2 = ethyl acetate extract, and 3 = ethanol extract.

In determining the TPC in each extract, gallic acid was used as standard, a linear regression equation of gallic acid was applied (*y* = 0.0056*x* + 0.0223, *R*^2^ = 0.998), and stated as the gallic acid equivalent. The TPC in crystal guava extracts presented various results from 1.82 to 49.55 g GAE/100 g (Figure 1). Ethanol leaves extract of crystal guava (LV3) showed the highest TPC (49.55 g GAE/100 g). Meanwhile, for the TFC, quercetin was utilized as standard. The linear regression of quercetin was *y* = 0.004*x* + 0.073; *R*^2^ = 0.991. This equation was applied in the investigation of the TFC in each extract. The TFC in leaves and fruit extracts from crystal guava was in the range of 0.18–9.68 g·QE/100 g. The highest flavonoid content (9.68 g·QE/100 g) was exposed by n-hexane leaves extract (LV1) (Figure 1).

The total phenolic content might be linked with antioxidant capacity. The highest TPC in the present study was given by ethanol leaves extract (49.55 g·GAE/100 g), while the TFC was given by n-hexane leaves extract (9.68 g QE/100 g). In the previous study [9], it was reported that the TPC of ethanol leaves extract (146.7 mg·GAE/g·dw) was higher than that of ethyl acetate extract (99.6 mg·GAE/g·dw). It was similar with the current study, as the TPC of ethanol extract (49.55 g·GAE/100 g) was also higher than ethyl acetate extract (8.49 g·GAE/100 g). In another research by Bouchoukh et al. [6], it was figured that the TPC and TFC in ethyl acetate extract of guava that was cultivated in Algeria were 931.15 *μ*g GAE/mg and 269.57 *μ*g QE/mg, respectively. In this research, the TPC (8.49 g·GAE/100 g) and TFC (7.68 g·QE/100 g) of ethyl acetate leaves extract were lower than those in the previous research. Ashraf et al. [7] reported that the TPC (83.34 *μ*g·GAE/mg extract) and TFC (53.39 *μ*g·QE/mg extract) of methanol leaves extract of Pakistan guava were higher than those of chloroform and n-hexane extracts. In the previous study [7], the TPC and TFC of n-hexane extract were 53.24 *μ*g·GAE/mg extract and 21.26 *μ*g·QE/mg extract, respectively. It was higher than our result because the TPC of n-hexane leaves extract was 3.39 g·GAE/100 g. Meanwhile, it was different for the TFC because in our study, n-hexane leaves extract gave higher result (9.68 g·QE/100 g) than ethyl acetate and ethanol leaves extracts. Research by Cedric et al. [10] revealed that the TPC and TFC of ethyl acetate leaves extract (15.328 mg GAE/g and 1.991 mg CE/mg) were higher than those of n-hexane extract. It was similar with our result because the TPC of ethyl acetate leaves extract was higher than n-hexane extract, but it was different for the TFC. The study by Liu et al. [11], using white-guava seed, demonstrated that the highest TPC and TFC were found in peel extract (39.65 mg·GAE/g·dw and 19.72 mg·RE/g·dw), compared to flesh and seed extracts.

### 3.3. Correlation between the TPC and TFC with AAI DPPH, CUPRAC, and FRAP in Crystal Guava Extracts

The highest AAI from each method gave the highest antioxidant capacity. Therefore, the TPC and TFC contributed to the antioxidant capacity, when the correlation was significant and positive (Table 2).

The TPC was significantly positively correlated with the AAI of all extracts (0.824 ≤ *r* ≤ 0.990). Meanwhile, the TFC was only significantly positively correlated with the AAI of fruit extract (0.924 ≤ *r* ≤ 0.996), where an increase in the TPC and/or TFC in fruit of crystal guava would increase the antioxidant activity by DPPH, CUPRAC, and FRAP methods, while in leaves extract, an increase in the TPC would increase the antioxidant activity by all three methods. In the previous study, condensed tannin in guava, perhaps, gave contribution to the antioxidant activity [10]. This result is also in agreement because condensed tannin is one of the phenolic compounds. Based on the current research, it could be suggested that the determination of the TPC and TFC in fruit extracts can be used to predict, indirectly, its antioxidant capacities by DPPH, CUPRAC, and FRAP methods, while those of leaves extract could be predicted indirectly only by determining their TPC. There were many studies that used IC_50_ or EC_50_ in their result, so higher TPC and/or TFC is related with higher antioxidant capacities which is shown by lower IC_50_ or EC_50_, when the correlation was negative and significant [23]. Similar with our result, Bouchoukh et al. [6] exposed that the TPC in leaves extract of *Psidium guajava* that is cultivated in Algeria gave a negative and significant correlation with their IC_50_ of DPPH and ABTS (*r* = −0.986, *r* = −0.982 *p* < 0.01, respectively).

Different structures of flavonoid and phenolic in samples could give different antioxidant activities. Higher antioxidant capacity was shown by a sample that had a phenolic compound with more hydroxyl groups or conjugated double bonds. A high antioxidant capacity in a flavonoid will be presented when the flavonoid has a hydroxyl group in C-3′-C-4′, OH at C-3, oxo function at C-4, double bond at C-2 and C-3 [24]. The highest effect in the antioxidant capacity of a flavonoid was possessed by the ortho position of the hydroxyl group in C-3′-C-4′ [25]. The TFC in FR1 was higher than the TFC in FR3, but AAI DPPH of FR3 was higher than FR1; therefore, most of the flavonoids in FR3 were estimated to have a hydroxyl group in C-3′-C-4′, OH at C-3, oxo function at C-4, double bond at C-2 and C-3, while most of the flavonoids in FR1 with other positions have low antioxidant activity. The TPC in FR1 (2.05 g·GAE/100 g) was higher than the TPC in FR3 (1.89 g·GAE/100 g), but AAI DPPH of FR3 (0.34) was similar with FR1 (0.33). It means that many phenolic compounds in FR3 have higher ability in transfer hydrogen than FR1. The TPC in FR2 (5.31 g·GAE/100 g) was lower than the TPC in LV2 (8.49 g·GAE/100 g), but AAI FRAP of FR2 (37.40) was higher than LV 2 (17.41). It can be suggested that the majority of phenolic compounds in FR2 have lower reduction potential than Fe (III)/Fe (II) 0.77 V.

Different mechanisms might give different antioxidant capacities. The correlation between three antioxidant assays in the current study has been analysed using Pearson's method (Table 3). The three methods showed different results among each other. Pearson's correlation could analyse whether the AAI of each method gave a linear result or not. One method will give a linear result with the other method when the correlation was positive and significant. It means one method and the other method could give different results, but the two methods could present the linear results. Based on data in Table 3, it can be seen that the tree methods in the present research gave linear results for leaves and fruit extract of crystal guava.

## 4. Conclusions

The antioxidant activity of samples should be investigated using different methods in parallel because it could be exposed as different results. All of ethyl acetate and ethanol leaves extracts of crystal guava were very strong antioxidants using DPPH, CUPRAC, and FRAP methods. For the highest antioxidant activity, ethanol was the best solvent for extraction leaves and ethyl acetate for extraction fruit of crystal guava. There was a positive and high correlation between the TPC in leaves extract with the AAI of DPPH, CUPRAC, and FRAP. The phenolic compound in crystal guava leaves mainly contributed to their antioxidant activity by DPPH, CUPRAC, and FRAP methods. DPPH, CUPRAC, and FRAP methods presented linear results in the antioxidant activity of leaves and fruit extracts. Crystal guava leaves may be developed as a natural antioxidant for the food and nutrition industry.

## Figures and Tables

**Table 1:** Antioxidant activities of crystal guava extracts by DPPH, FRAP, and CUPRAC assays.

Sample	*AAI DPPH*		
	Leaves	Fruit	Ascorbic acid
n-Hexane extract	1.0007 ± 0.0270^a^	0.3391 ± 0.0110^a^	29.4561 ± 1.573
Ethyl acetate extract	1.8259 ± 0.0700^a^	0.5927 ± 0.0290^b^	
Ethanol extract	56.4614 ± 3.7810^b^	0.3493 ± 0.0120^a^	

	*AAI FRAP*		

n-Hexane extract	3.668 ± 0.0350^a^	2.910 ± 0.0197^a^	92.911 ± 4.526
Ethyl acetate extract	17.413 ± 0.0577^b^	37.405 ± 0.7065^b^	
Ethanol extract	59.894 ± 0.5286^c^	1.658 ± 0.0079^c^	

	*AAI CUPRAC*		

n-Hexane extract	0.952 ± 0.0094^a^	0.725 ± 0.0048^a^	21.988 ± 1.8726
Ethyl acetate extract	2.848 ± 0.0107^b^	1.512 ± 0.0057^b^	
Ethanol extract	7.312 ± 0.0378^c^	0.204 ± 0.0008^c^	

a–c = different letters in the same column show the significant difference (*p* < 0.05).

**Table 2:** Correlation of the TPC and TFC of crystal guava extracts with AAI DPPH, CUPRAC, and FRAP.

Antioxidant parameter	Pearson's correlation coefficient (*r*)	
	TPC	TFC
AAI DPPH LV	0.824^*∗∗*^	−0.900^*∗∗*^
AAI DPPH FR	0.982^*∗∗*^	0.924^*∗∗*^
AAI FRAP LV	0.990^*∗∗*^	−0.969^*∗∗*^
AAI FRAP FR	0.987^*∗∗*^	0.948^*∗∗*^
AAI CUPRAC LV	0.981^*∗∗*^	−0.979^*∗∗*^
AAI CUPRAC FR	0.946^*∗∗*^	0.996^*∗∗*^

^*∗∗*^ = significant at *p* < 0.01.

**Table 3:** Correlation between AAI DPPH, CUPRAC, and FRAP.

Antioxidant parameter	Pearson's correlation coefficient (r)					
	AAI FRAP LV	AAI FRAP FR	AAI CUPRAC LV	AAI CUPRAC FR	AAI DPPH LV	AAI DPPH FR
AAI DPPH LV	0.973^*∗∗*^					
AAI DPPH FR		0.991^*∗∗*^				
AAI FRAP LV			0.998^*∗∗*^			
AAI FRAP FR				0.930^*∗∗*^		
AAI CUPRAC LV					0.959^*∗∗*^	
AAI CUPRAC FR						0.896^*∗∗*^

^*∗∗*^ = significant at *p* < 0.01.

## Data Availability

The data used to support the findings of this study are available from the corresponding author upon request (hnadifan@gmail.com).
